# Suicide risk in patients with a current depressive episode during the COVID-19 pandemic

**DOI:** 10.3389/fpsyt.2024.1343323

**Published:** 2024-04-19

**Authors:** Mikhail Zinchuk, Georgii Kustov, Massimiliano Beghi, Yulia Bryzgalova, Ekaterina Sviatskaia, Sofya Popova, Nadezhda Voinova, Marina Terentieva, Alexander Yakovlev, Alla Guekht

**Affiliations:** ^1^Moscow Research and Clinical Center for Neuropsychiatry, Moscow, Russia; ^2^Department of Mental Health, azienda unità sanitaria locale (AUSL) Romagna, Cesena, Italy; ^3^Institute of Higher Nervous Activity and Neurophysiology, Russian Academy of Sciences, Moscow, Russia; ^4^Department of Neurology, Neurosurgery and Medical Genetics, Pirogov Russian National Research Medical University, Moscow, Russia

**Keywords:** COVID-19, major depressive disorder, MDD, suicide, NSSI, anxiety, Russia, self-harm

## Abstract

**Introduction:**

The prevalence of major depressive disorder (MDD) increased during the COVID-19 pandemic. Data on suicidality in these patients during the pandemic period remain scarce. The aim of the study was to determine the prevalence and variables associated with serious suicide risk in Russian inpatients with MDD during the COVID-19 pandemic.

**Methods:**

A cross-sectional cohort study with consecutive sampling was conducted from January 1, 2021 to December 31, 2021. All patients completed the Mini International Neuropsychiatric Interview (M.I.N.I.) (including the suicidality module), the Beck Depression Inventory, and the State-Trait Anxiety Inventory, and underwent a semi-structured interview to collect relevant demographic and clinical data. Effect sizes for all independent variables and covariates were calculated using partial eta-squared (ηp2).

**Results:**

Of the 6757 patients with non-psychotic mental disorders assessed, 1605 (23.7%) had MDD confirmed by the M.I.N.I., of whom 17.8% were at serious risk for suicide according to the M.I.N.I. suicidality module. Factors independently associated with serious suicide risk in Russian inpatients with MDD during the pandemic were younger age (ηp2 = 0.021), greater severity of depression (0.038), higher state anxiety (0.003), and nonsuicidal self-injury (NSSI) (0.066). The same variables, except for state anxiety, were independently associated with suicide risk in the subgroup of MDD patients previously infected with SARS-CoV2.

**Conclusion:**

In the COVID-19 pandemic, the proportion of patients with MDD at serious risk of suicide was similar to pre-pandemic data. No associations were found between suicidality in patients with MDD and COVID-related factors. Younger age, greater severity of depression, and especially NSSI were the most significant risk factors for suicide in patients with MDD during the COVID-19 pandemic.

## Introduction

The coronavirus disease 2019 (COVID-19) pandemic has had a severe impact on the mental health of people around the world (1, 2). For example, the recent Global Burden of Disease (GBD) study estimated 53.2 million additional cases of major depressive disorder (MDD) globally due to the COVID-19 pandemic (3). Since the beginning of the pandemic, the increase in suicide rates has been predicted by experts in the field of self-injurious behavior (4, 5).

These concerns were based not only on the increase in rates of MDD, but also on a prosuicidal effect identified in pre-pandemic studies for factors such as job loss, social isolation, bereavement, domestic violence and disruption of mental health services. The dramatic increase in the prevalence of these problems was reported in studies conducted after the outbreak of the pandemic (6–10). In addition, the SARS-CoV-2 virus affects several biological processes that may be involved in the development of suicidal behavior (e.g., the rennin-angiotensin system, nicotinic receptors, and central and systemic inflammation) (11, 12).

Data on suicidality in the first year of the pandemic are conflicting. Increases in suicide deaths have been reported in some countries (13–15), and several meta-analyses and systematic reviews have found an increased prevalence of suicidal ideation (SI), suicide attempts (SA) and self-harm during the pandemic (16–20). At the same time, contrary to initial fears, suicide rates have remained stable in many regions, and in some have even declined from pre-pandemic levels (21). These results should be interpreted with caution because the vast majority of studies have been conducted in the general population of high-income countries, while the population most at risk of death by suicide - people with severe mental disorders from low- and middle-income countries - remains understudied (22).

A recent study of psychiatric inpatients showed a higher frequency of SA compared with patients admitted before the COVID-19 pandemic (23). In the study by Perlis et al. (2021), higher levels of suicidality were found in MDD patients with a COVID-19 history (24). Meanwhile, studies on this topic remain scarce and their results need to be reproduced in different populations to test hypotheses about the effect SARS-CoV2 infection on suicide risk in people with mental disorders.

Historically, self-harm has been a significant problem in Russia (25, 26), and over the past few decades, the suicide mortality rate in the Russian Federation has been consistently higher than the average for the geographic region. For example, according to WHO, the crude suicide rate for men (all ages) in Russia in 2019 was 43.6 per 100 000.

According to recent studies, a number of demographic and clinical characteristics distinguish Russian suicide ideators and attempters from those living in other European countries (27). These data contrasted with a relatively low level of research interest in the problem: despite having the highest male suicide rate among the G8 countries, Russia had the fewest published papers and the least collaboration with other G8 countries in this area (28).

A study directly addressing the issue of suicidality in the early months of the COVID-19 pandemic found no increase in suicide rates in St Petersburg (the second most populous city in Russia after Moscow) (21). At the same time, the international COVID-19 study of mental health in the general population (COMET-G), with the highest proportion of participants from Russia, found that suicidality was increased in people with a history of mental disorders (RR for manifestation of at least moderate SI was 13.5 for those with a history of psychotic disorder and 7.37 for those with a history of non-psychotic disorder) (29). To date, there is a lack of data on suicidality in Russian MDD patients during the COVID-19 pandemic.

The primary objective of the study was to determine the prevalence of serious suicide risk in Russian inpatients with MDD during the COVID-19 pandemic. Secondary objectives were to determine variables associated with serious suicide risk in patients with MDD and to test the hypothesis that SARS-CoV2 infection or some COVID-19 disease characteristics increase suicide risk in patients with MDD.

## Patients and methods

### Study design

Our research was designed as a cross-sectional cohort study with consecutive sampling. In the period from January 1, 2021 to December 31, 2021, all patients admitted to the Moscow Research and Clinical Center of Neuropsychiatry (MRCCN) were evaluated to identify those who met the inclusion criteria of the study.

### Setting

In the Russian Federation, people with non-psychotic mental disorders are treated separately from those who have developed psychosis and those whose primary problem is substance addiction. The MRCCN is the largest center for patients with non-psychotic mental disorders in Moscow - Russia’s most populous city (13010112 officially registered residents in the year of the study). Anyone with a permanent registration in Moscow who is experiencing mental health problems can either come to the center on their own or be referred for psychiatric help by a general practitioner. The cost of the treatment is covered by the Moscow Healthcare Department and is therefore free of charge to the patient.

### Participants

The study sample represented all patients (n=6757) referred to the center’s inpatient psychiatric unit for a non-psychotic mental disorder requiring medical intervention. On admission, adult patients (≥ 18 years) were screened for cognitive impairment using the Mini-Mental State Examination (MMSE) (30). Patients without evidence of cognitive impairment (MMSE score > 24) were interviewed using the Russian version of the Mini International Neuropsychiatric Interview (M.I.N.I.) (31) version 7.0.2.

The population of interest in our study was patients with current MDD as assessed by the M.I.N.I. Non-inclusion criteria were a lifetime history of manic/hypomanic episodes, a history of psychotic symptoms not related to an affective disorder, and cognitive impairment that made it impossible to understand the interviewer’s questions and complete the questionnaires. Patients vaccinated against COVID-19 were not included in the analysis, even if they were infected with SARS-CoV2 prior to vaccination. Recent reports suggest that some may be susceptible to neurological (32) and psychiatric (33) events following vaccination. All patients with MDD were divided into two groups: low suicide risk and serious suicide risk.

### Variables

The outcomes of interest were 12-month SI, SA, nonsuicidal self-injury (NSSI), and serious suicide risk. In our study, SI was defined as thoughts of killing oneself, SA - an actual attempt to kill oneself with at least some intent to die, and NSSI - hurting oneself without wanting to die (34). Moderate to high suicide risk (scores ≥ 9) on the M.I.N.I. was considered serious suicide risk, while low suicide risk was defined as those scoring less than 9.

Based on the results of previous studies, we proposed that the following factors might be associated with serious suicide risk: sex assigned at birth, age, education level, marital status, employment status, additional mental disorder diagnosis, level of anxiety, and level of depression. The COVID-19-related factors that were assessed for association with outcomes of interest were: history of COVID-19 infection, time after COVID-19, type of care for COVID-19, and acute COVID-19 symptoms.

### Data sources/measurement

The M.I.N.I. is a structured diagnostic interview designed to assess the most common mental disorders and is considered by many researchers to be the gold standard in its field. The Suicidality Module of the M.I.N.I. focuses mainly on the current SI and SA (last 4 weeks) and allows a grading of the suicide risk ranging from low (range is between 1 and 8 points), to intermediate (9 - 16), and high risk (≥17). Moderate to high suicide risk (scores ≥ 9 points) was considered a serious suicide risk, while low suicide risk was defined as those with scores less than 9.

All patients with MDD underwent an *ad hoc* semi-structured interview to collect demographic (age, sex assigned at birth, educational level, marital and employment status) and clinical data (lifetime and 12-month history of SI, SA and NSSI) and completed two self-report inventories to measure levels of depression (Beck Depression Inventory (BDI)) and anxiety (State-Trait Anxiety Inventory (STAI)). The BDI (35) is a 21-item multiple-choice, self-report inventory that is one of the most widely used instruments for measuring the severity of depression. The STAI (36) is a commonly used measure of trait (STAI-T) and state (STAI-S) anxiety (20 items assessing trait anxiety and 20 items assessing state anxiety). Both instruments had previously been validated in the Russian-speaking population (37, 38) and showed psychometric properties close to the original versions.

SARS-CoV2 infection was evidenced by assessment of IgG-specific antibody titers. Titer levels were assessed on the day of admission via a chemiluminescence immunoassay. For those who were previously diagnosed with a SARS-CoV2 infection all COVID-19-related information (e.g. date of diagnosis, symptoms, type of care, PCR test result, etc.) was extracted from the Moscow “Unified medical data-analytical system” (EMIAS). This web-based information system contains information on all cases of the health service utilization (medical check-ups, inpatient, outpatient and emergency medical care) for all citizens of Moscow. In addition, all patients who developed symptoms in the acute phase of SARS-CoV2 infection were assessed using the first module, the Russian version of the standardized Case Report Form for Post COVID condition (Post COVID-19 CRF). This tool has been developed by the World Health Organization (39) to report standardized clinical data from individuals following COVID-19. The first module of the Post COVID-19 CRF is designed to collect detailed clinical information about the acute episode of COVID-19.

### Statistical analysis

Descriptive statistics of demographic and clinical variables are presented as “mean (standard deviation)” for continuous variables and as “N (%)” for categorical variables, and the prevalence of SI, SA, NSSI and serious suicide risk is presented as “% (95% confidence interval (CI))”.

A series of univariate analyses of serious suicide risk with demographic and clinical variables were performed in the whole sample (sex assigned at birth, age, current marital status, level of education, comorbid mental disorder, NSSI, BDI, STAI-S, STAI-T and SARS-CoV2 infection) and in the group of patients with a COVID-19 history (time after COVID-19, type of care for COVID-19, acute COVID-19 symptoms). Categorical variables were compared using Pearson’s chi-squared test and continuous variables were compared using the Mann-Whitney U test. The Benjamini-Hochberg correction was used to correct for multiple comparisons for additional diagnoses of mental disorders and acute COVID-19 symptom variables (40, 41).

Variables with a p-value of less than <.05 were entered into a series of general linear models to identify independent predictors of serious suicide risk in the overall sample and in patients with a history of COVID-19. Serious suicide risk was entered as the dependent variable in separate models, with categorical clinical and demographic variables as independent variables and continuous variables as covariates. Effect sizes for all independent variables and covariates were calculated using partial eta-squared (ηp2). Adjusted R-squared was calculated to assess the strength of association between the dependent variables and the predictor variables. All tests were two-tailed and statistical significance was set at p <.05.

Variables with a component frequency of less than 3% were excluded from the analysis.

Statistical analysis was performed using the jamovi program v 2.3.17 (42). Descriptive statistics were calculated using jamovi’s exploration module, 95% CI in the ESCI module, Mann-Whitney U test in the T-test module, Fisher’s exact test in the frequencies module and GAMLJ module for the general linear model.

This study adhered to the tenets of the Declaration of Helsinki. The study protocol was approved by the local research ethics committee of the MRCCN (protocol number 46). Written informed consent was obtained from all patients prior to any study procedures. This work was supported in part by the Moscow Center for Healthcare Innovations [grant number 1108-1/22 from 05.04.2022].

## Results

### Participants

During the study period, 7354 patients with non-psychotic mental disorders were referred to the MRCCN for inpatient treatment, of whom 597 were not included in the study because of being vaccinated, had an MMSE score <24, refused to participate in the study or completed questionnaires inappropriately. Of the remaining 6757 patients, 1605 (23.7%) were diagnosed with MDD.

### Descriptive data

The mean age of the participants was 46.9 (SD 15.1: min-max 18-73) years. Most of them were assigned female at birth (1256 (78%)). Almost half of the patients were in a relationship or married (710 (45.5%)). Eight hundred and two (50.1%) patients had a higher education and 600 (37.4%) were employed or students.

According to M.I.N.I., 1090 (67.9%) patients were diagnosed with a comorbid mental disorder, including anxiety disorders in 975 (60.7%), obsessive-compulsive disorder in 135 (8.4%), post-traumatic stress disorder in 80 (5.0%), alcohol abuse in 67 (4.2%), substance abuse in 22 (1.4%), eating disorders in 80 (5.0%) and antisocial personality disorder in 21 (1.3%).

About half of the patients were infected with SARS-CoV2 (844 (52.6%)), of which 625 (74.1%) reported at least one COVID-19 acute-phase symptom. Of these, the majority of patients (625 (74.1%)) had coronavirus infection with symptoms in the acute phase. At the same time, 77 (9.1%) people were treated in hospital and 55 (6.5%) people underwent resuscitation.

### Suicidality and nonsuicidal self-injurious behavior

The lifetime prevalence of SI was 34.3% (95% CI 32.0-36.6%), SA was 12.2% (95% CI 10.7-13.9%) and NSSI was 14.1% (95% CI 12.5-15.9%).

In the past 12 months, 28.5% (95% CI 26.4-30.8%) of the sample had SI, 4.1% (95% CI 3.2-5.1%) had attempted suicide and 8.4% (95% CI 7.2-9.9%) had NSSI.

According to the results of the M.I.N.I. Suicide Module, 17.8% (95% CI 16.0-19.7%) of patients with MDD were at serious risk for suicide (5.11% were at moderate risk and 12.65% were at severe risk for suicide).

### Factors associated with serious suicide risk in the entire sample

Factors associated with moderate/severe suicide risk in the univariate analysis were: age, employment status, marital status, comorbid obsessive-compulsive disorder, alcohol use disorder, eating disorders, lifetime NSSI, BDI, STAI-S, STAI-T scores and negative COVID-19 history (**Table 1**).

**Table 1:** Factors associated with serious suicide risk in the entire sample.

	Mean (SD) or number (%)		*p-value*
	Moderate/severe SR	No/low SR	
	N=285	N=1320	
Sex assigned at birth			
*Male*	58 (20.4 %)	291 (22.0 %)	0.525 χ2 = 0.396 df=1
*Female*	227 (79.6 %)	1029 (78.0 %)	
Age			
*18-29*	124 (43.5 %)	153 (11.6 %)	<0.001 χ2 = 192.339 df=4
*30-39*	53 (18.6 %)	206 (15.6 %)	
*40-49*	32 (11.2 %)	246 (18.6 %)	
*50-59*	51 (17.9 %)	310 (23.5 %)	
*≥60*	25 (8.8 %)	405 (30.7 %)	
Current marital status			
*Married*	80 (28.1 %)	548 (41.5 %)	<0.001 χ2 = 0.396 df=1
*Widowed*	15 (5.3 %)	159 (12.1 %)	
*Divorced*	51 (17. 9 %)	291 (22.1 %)	
*Single*	116 (40.6 %)	243 (18.3 %)	
*In an unregistered relationship*	23 (8.1 %)	79 (6.0 %)	
Highest level of education			
*Higher*	129 (45.3 %)	675 (51.1 %)	<0.001 χ2 = 76.350 df=3
*Incomplete higher*	59 (20.7 %)	108 (8.2 %)	
*Specialised secondary*	56 (19.7 %)	414 (31.4 %)	
*High/Secondary school*	41 (14.4 %)	123 (9.3 %)	
Employment status			
*In education/training*	35 (12.3 %)	35 (2.7 %)	<0.001 χ2 = 85.932 df=3
*Employed*	102 (35.8 %)	428 (32.4 %)	
*Not currently employed*	108 (37.9 %)	404 (30.6 %)	
*Retired/unable to work*	40 (14.0 %)	453 (34.3 %)	
Additional diagnosis of mental disorder			
*Anxiety disorders*	181 (63.5 %)	794 (60.2 %)	0.293 χ2 = 1.108 df=1
*Obsessive-compulsive disorder*	39 (13.7 %)	96 (7.3 %)	<0.001* χ2 = 12.507 df=1
*Post-traumatic stress disorder*	17 (6.0 %)	63 (4.8 %)	0.401 χ2 = 0.703 df=1
*Alcohol use disorder*	22 (7.7 %)	45 (3.4 %)	<0.001* χ2 = 10.886 df=1
*Eating disorders*	32 (11.2 %)	48 (3.6 %)	<0.001* χ2 = 28.524 df=1
NSSI	127 (44.6 %)	100 (7.6%)	<0.001 χ2 = 264.049 df=1
BDI	32.68 (8.6)	26.71 (7.4)	<0.001 U=113799.500
STAI-S	61.49 (8.7)	59.66 (10.0)	0.010 U=168461.500
STAI-T	62.14 (9.2)	58.18 (9.2)	<0.001 U=140495.000
COVID-19 exposure	108 (37.9 %)	736 (55.8 %)	<0.001 χ2 = 29.996 df=1

*Remained significant after Benjamini-Hochberg correction; SR, Suicide risk; NSSI, Nonsuicidal self-injurious behavior; BDI, Beck Depression Inventory; STAI-S, State-Trait Anxiety Inventory – State anxiety; STAI-T, State-Trait Anxiety Inventory – Trait anxiety.

The comparison of the BDI, STAI-S and STAI-T scores were based on Mann–Whitney U test and the other variables were compared with chi-square test.

In the multivariate analysis, factors that remained significant were age (ɳ2 = 0.021, p<0.001), lifetime NSSI (ɳ2 = 0.066, p<0.001), BDI (ɳ2 = 0.038, p<0.001) and STAI-S (ɳ2 = 0.003, p=0.022) scores. All factors included in the multivariate model accounted for 23.5% (R2 = 0.235) of the variance in predicting serious suicide risk in patients with MDD (**Table 2**).

**Table 2:** General linear model for suicide risk in the total sample.

Variable	Partial eta-squared	df	p-value	df	AdjR^2^	p-value
Age	0.021	4	< 0.001	21	0.235	< 0.001
Marital status	0.004	4	0.168			
Education	0.001	3	0.725			
Employment status	0.001	3	0.389			
Obsessive-compulsive disorder	0	1	0.666			
Alcohol abuse	0.002	1	0.081			
Eating disorders	0.001	1	0.251			
NSSI	0.066	1	<0.001			
BDI score	0.038	1	< 0.001			
STAI-S score	0.003	1	0.022			
STAI-T score	0.001	1	0.24			
COVID-19 exposure	0.002	1	0.055			

df, degrees of freedom; AdjR^2^, adjusted R^2^; NSSI, Nonsuicidal self-injurious behavior; BDI, Beck Depression Inventory; STAI-S, State-Trait Anxiety Inventory – State anxiety; STAI-T, State-Trait Anxiety Inventory – Trait anxiety.

### Factors associated with serious suicide risk in patients with a history of SARS-CoV-2 infection

In the univariate analysis (patients with a history of SARS-CoV-2 infection only), serious suicide risk in MDD patients was associated with age, employment status, marital status, comorbid obsessive-compulsive disorder, alcohol use disorder, eating disorders, lifetime NSSI, BDI, STAI-S, STAI-T scores and type of care for COVID-19 (**Table 3**). No significant difference was found between MDD patients with and without serious suicide risk with regard to other COVID-related variables.

**Table 3:** Factors associated with serious suicide risk in the COVID-19 sample.

	Mean (SD) or number (%)		*p-value*
	Moderate/severe SR	No/low SR	
	N=108	N=736	
Sex assigned at birth			
*Male*	21 (19.4 %)	156 (21.2 %)	0.671 χ2 = 0.174 df=1
*Female*	87 (80.6 %)	579 (78.8 %)	
Age			
*18-29*	46 (42.6 %)	68 (9.2 %)	<0.001 χ2 = 99.332 df=4
*30-39*	15 (13.9 %)	109 (14.8 %)	
*40-49*	8 (7.4 %)	131 (17.8 %)	
*50-59*	28 (25.9 %)	191 (26.0 %)	
*≥60*	11 (10.2 %)	237 (32.2 %)	
Current marital status			
*Married*	30 (27.8%)	327 (44.4 %)	<0.001 χ2 = 30.284 df=4
*Widowed*	7 (6.5%)	90 (12.2 %)	
*Divorced*	25 (23.1%)	161 (21.9 %)	
*Single*	39 (36.1%)	116 (15.8 %)	
*In an unregistered relationship*	7 (6.5%)	42 (5.7 %)	
Highest level of education			
*Higher*	56 (51.9 %)	383 (52.0 %)	<0.001 χ2 = 19.903 df=3
*Incomplete higher*	19 (17.6 %)	48 (6.5 %)	
*Specialized secondary*	21 (19.4 %)	237 (32.2 %)	
*High/Secondary school*	12 (11.1 %)	68 (9.2 %)	
Employment status			
*In education/training*	47 (43.5 %)	252 (34.2 %)	<0.001 χ2 = 51.697 df=3
*Employed*	36 (33.3 %)	215 (29.2 %)	
*Not currently employed*	13 (12.0 %)	13 (1.8 %)	
*Retired/unable to work*	12 (11.1 %)	256 (34.8 %)	
Additional diagnosis of mental disorder			
*Anxiety disorders*	69 (63.9%)	436 (59.2%)	0.357 χ2 = 0.847 df=1
*Obsessive-compulsive disorder*	9 (8.3%)	45 (6.1%)	0.379 χ2 = 0.774 df=1
*Post-traumatic stress disorder*	3 (2.8%)	34 (4.6%)	0.383 χ2 = 0.762 df=1
*Alcohol use disorder*	4 (3.7%)	17 (2.3%)	0.385 χ2 = 0.754 df=1
*Eating disorders*	11 (10.2%)	15 (2.0%)	<0.001* χ2 = 20.938 df=1
NSSI	42 (38.9%)	40 (5.4%)	<0.001 χ2 = 120.164 df=1
BDI	31.81 (8.84)	25.55 (7.78)	<0.001 U=23856.000
STAI-S	61.34 (8.68)	58.78 (10.30)	0.016 U=33432.000
STAI-T	61.49 (9.53)	57.67 (9.05)	<0.001 U=30238.500
Time after COVID-19			
*Unknown*	4 (3.7 %)	46 (6.3 %)	0.743 χ2 = 1.240 df=3
*<3 months*	17 (15.7 %)	119 (16.2 %)	
*3-6 months*	36 (33.3 %)	246 (33.4 %)	
*>6 month*	51 (47.2 %)	325 (44.2 %)	
Type of care for COVID-19			
*Home treatment*	101 (93.5 %)	611 (83.0 %)	0.017 χ2 = 8.108 df=2
*Hospitalization (non-ICU care)*	5 (4.6 %)	72 (9.8 %)	
*Hospitalization (ICU)*	2 (1.9 %)	53 (7.2 %)	
**Acute COVID-19 symptoms**	81 (75.0 %)	544 (73.2 %)	0.810 χ2 = 0.058 df=1
Fever	45 (41.7 %)	291 (39.5 %)	0.673 χ2 = 0.278 df=1
Cough	44 (40.7 %)	281 (38.2 %)	0.609 χ2 = 0.261 df=1
shortness of breath	35 (32.4 %)	199 (27.0 %)	0.244 χ2 = 1.355 df=1
reduced smell/taste	55 (50.9 %)	333 (45.2 %)	0.269 χ2 = 1.224 df=1
headache	48 (44.4 %)	327 (44.4 %)	0.998 χ2 = 0.000 df=1
muscle pain	45 (41.7 %)	299 (40.6 %)	0.837 χ2 = 0.042 df=1
dizziness	31 (28.7 %)	206 (28.0 %)	0.877 χ2 = 0.024 df=1
difficulty concentrating	27 (25.0 %)	178 (24.2 %)	0.854 χ2 = 0.034 df=1
increased sensitivity to light, sound or touch	25 (23.2 %)	144 (19.6 %)	0.385 χ2 = 0.755 df=1
diarrhea	12 (11.1 %)	109 (14.8 %)	0.306 χ2 = 1.049 df=1
other	14 (13.0 %)	108 (14.7 %)	0.637 χ2 = 0.223 df=1

*Remained significant after Benjamini-Hochberg correction; SR, suicide risk; NSSI, Nonsuicidal self-injurious behavior; BDI, Beck Depression Inventory; STAI-S, State-Trait Anxiety Inventory – State anxiety; STAI-T, State-Trait Anxiety Inventory – Trait anxiety.

Comparison of age (years), BDI, STAI-S and STAI-T scores was based on the Mann-Whitney U test, and the other variables were compared using the chi-squared test.

In the multivariate analysis, age (ɳ2 = 0.029, p<0.001), NSSI history (ɳ2 = 0.061, p<0.001) and BDI score (ɳ2 = 0.029, p<0.027) retained their significance. The overall model fit was adjusted R2 = 0.209, meaning that the factors in the model accounted for 20.9% of the variance in moderate/severe suicide risk (**Table 4**).

**Table 4:** General linear model for serious suicide risk in COVID-19 patients.

Variable	Partial eta-squared	df	p-value	df	AdjR^2^	p-value
Age	0.029	4	< .001	20	0.209	< 0.001
Marital status	0.01	4	0.085			
Education	0.001	3	0.796			
Employment status	0.007	3	0.117			
Eating disorders	0.001	1	0.433			
NSSI	0.061	1	< .001			
BDI score	0.027	1	< .001			
STAI-S score	0.003	1	0.132			
STAI-T score	0.001	1	0.455			
Care type for COVID-19	0.003	1	0.247			

df, degrees of freedom; AdjR^2^, adjusted R^2^; NSSI, Nonsuicidal self-injurious behavior; BDI, Beck Depression Inventory; STAI-S, State-Trait Anxiety Inventory – State anxiety; STAI-T, State-Trait Anxiety Inventory – Trait anxiety.

## Discussion

Despite a recent decline in suicide mortality in the Russian Federation (43) following changes in government alcohol policy (44), pre-pandemic suicide rates in the country remained higher than the European regional average (45). Rates of depression in the Russian general population were also high before the pandemic. According to the multicenter epidemiological survey ESSE-RF, 25.6% of Russian citizens aged 25-64 suffered from subclinical depression and 8.8% were found to have clinical depression (46). In addition, the proportion of people with MDD receiving treatment in Russia is likely to be lower than in other developed countries (47). If so, untreated depression may be one of the causes of the high suicide rate in the Russian Federation. There is a gap in knowledge about the prevalence of suicidality in Russian patients with MDD. Data from pre-pandemic studies may be biased by small study samples, diagnosis based on expert opinion, and lack of consensus among researchers on the definition of suicidality.

Despite conflicting data on the prevalence of suicidal symptoms in MDD patients across cohorts (48), most researchers now agree that MDD is a major risk factor for suicidality. In the study by Cai and colleagues, compared to non-MDD controls, the odds ratios (ORs) for lifetime and past-year SI in MDD were 2.9 and 14.0, respectively, while the ORs for lifetime and past-year prevalence of SA were 3. 5 and 7.3, respectively (49).

In our sample, 28.5% of participants had suicidal thoughts in the previous 12 months and 4.1% had attempted suicide. These levels are lower than those found in previous meta-analyses that did not include studies conducted in Russia. In the study by Cai and colleagues (2021), the overall prevalence of SI was 37.7% (50). According to the meta-analysis by Dong et al. (2019), the 1-year prevalence of SA was 8% (51). A meta-analysis including studies conducted in China found a 1-month prevalence of SI and SA of 27.7% and 20.3%, respectively (52).

On the one hand, our results are consistent with studies reporting a decrease in suicidality during the pandemic. For example, López-Moríñigo and colleagues found an inverse correlation between previous cross-national suicide rates and cumulative COVID-19 cases in 174 countries (53). However, these data should be extrapolated with caution due to the above-mentioned lack of knowledge about the prevalence of suicidality in Russian patients with MDD in the pre-pandemic period.

On the other hand, our results may be partly explained by the tool we used to assess suicide risk in patients. We suspect that researchers using a more sensitive and less specific tool than the M.I.N.I. interview might find a higher proportion of participants at risk of suicide.

MDD patients with moderate/high suicide risk in our study were significantly younger than those with low/no suicide risk. This may explain many of the socio-demographic differences between the groups. In addition, other authors confirm that lockdown (and especially distance learning) had the worst effects on the youngest, worsening their mood state (54, 55). In the general linear model for suicide risk, only 4 variables remained significant: age, severity of depression (BDI score), severity of state anxiety (STAI-S score) and NSSI behavior. In our study sample, the prevalence of NSSI was 14.1%. Most studies of the prevalence of NSSI have been conducted in the general population. For example, in a study of a representative sample of the German population, 3.1% reported having experienced NSSI at least once in their lifetime. The prevalence of NSSI is expected to be higher in the clinical population, as psychiatric disorders, including MDD, have been shown to be predictors of NSSI (56–58). At the same time, few studies have directly addressed the prevalence of NSSI in MDD and, to the best of our knowledge, the prevalence of NSSI in Russian patients with MDD has not been previously assessed. According to Kang and colleagues (2021), 34.2% of Chinese students with moderate to major depressive disorder had a lifetime history of NSSI (59). The prevalence of NSSI in our study is lower, which may be due to the fact that our study was not limited to a group of young patients, among whom NSSI is more common according to many studies.

In a general linear model for serious suicide risk, NSSI had the highest ηp2 among all variables examined. Our data support the hypothesis that NSSI is involved in the transition from suicidal ideation to suicide attempt by reducing the fear of committing suicide through the regular experience of pain and habitual viewing of blood associated with NSSI (60). A recent study from the Russian Federation reported that about 60% of inpatients with non-psychotic mental disorders and SI had a lifetime history of NSSI (61). Another study from Russia, conducted in patients with epilepsy, also reported an association between NSSI and both SI and SA (62).

Our findings of a higher prevalence of SI and nonfatal attempts among younger patients are consistent with previous findings (63), including studies of Russian patients with non-psychotic mental disorders (64, 65). Other factors independently associated with serious suicide risk in Russian patients with MDD during the pandemic were severity of depression and state anxiety. These data are consistent with the results of the pre-pandemic studies. For example, a study by Hawton and colleagues (2013) found that suicide in people with depression was associated with more severe depression (OR=2.20) and anxiety (OR=1.59) (66). A recent study by Guerrero-Barona and colleagues (2021), also using the BDI and STAI, also found an association between high scores on these questionnaires and suicidality (67). In the aforementioned study, more significant correlations with suicidality were found for state anxiety than for trait anxiety.

The study results do not support the hypothesis (68) that SARS-CoV2-infected individuals are at higher risk for suicide because of some of the underlying mechanisms shared with COVID-19. Furthermore, no associations were found between serious suicide risk in patients with MDD and COVID-19 characteristics, including acute-phase symptoms. Risk factors for serious suicidality in MDD with a history of SARS-CoV2 infection were quite similar to those we found for the whole sample (younger age, BDI score, NSSI), except for the STAI-S score, which was significant in the multivariate analysis.

### Significance of the study

Our study adds data from Russian people with MDD to the existing body of knowledge on factors associated with suicidality in high-risk clinical populations, such as people with mental disorders. It fills several important research gaps by: 1. determining the prevalence of MDD in Russian patients admitted to psychiatric hospitals; 2. determining the proportion of patients with serious suicide risk in the population of patients with MDD during the pandemic; 3. determining variables associated with serious suicide risk in patients with MDD during the pandemic; 4. assessing an impact of SARS-CoV2-related parameters on suicide risk in patients with MDD. We found several parameters associated with serious suicide risk that could be used to identify a high-risk subgroup of patients with MDD who need to be carefully assessed for the presence of suicidality. In addition, three of the four independent factors associated with suicide risk are potentially modifiable (state anxiety level, depression severity, and NSSI) and could therefore be the intervention targets of suicide prevention protocols for inpatients with MDD. The highest ηp2 of NSSI among all variables associated with suicide risk supports the hypothesis that NSSI is a highly significant clinical phenomenon because of its involvement in the transition from suicidal ideation to suicide attempt in high suicide risk populations.

### Limitations

The main strength is the study of a population at high risk of suicide during the pandemic, for which information on predictors is lacking. As we used instruments that have been validated in many countries (the M.I.N.I., BDI, STAI and Post COVID-19 CRF), the methodology of our research can be easily reproduced. Such studies will allow comparison of data from different populations and provide an understanding of cultural specificities in the development of suicidality in patients with MDD. Another strength is the enrolment of consecutive patients to minimize selection bias.

There are, however, several limitations. The first is the relatively small number of patients with a severe course of COVID-19 in our sample. The severity of COVID-19 may be positively correlated with the severity of mental health outcomes, including suicidality. For example, in the study by Taquet and colleagues, higher hazard ratios for the development of neurological and mental disorders were reported in patients with more severe COVID-19 (admitted to intensive care compared with those who were not admitted) (69). The small number of patients with severe COVID-19 course may be related to the predominance of relatively young patients in our sample. In the meta-analysis by Katzenschlager and colleagues (2021), older age was associated with an increased risk of severe coronavirus infection and consequently the need for inpatient treatment, including intensive care (70).

The second limitation is the baseline characteristics of the cohort. Our participants were admitted to the Center for Neuropsychiatry for inpatient treatment of psychiatric symptoms severe enough to require hospitalization. There is a preponderance of individuals assigned female at birth in our sample is consistent with data from previous studies on their greater use of psychiatric care, in contrast to the underutilization of psychiatric services by individuals with mental disorders assigned male at birth. A recent study by Zinchuk and colleagues (2023) evaluating data from both the last pre-pandemic and the first year of the COVID-19 outbreak found that over 76% of patients at the Moscow clinic for patients with non-psychotic mental disorders are women (71). Therefore, conclusions from our study should be extrapolated with caution to individuals assigned male at birth. In addition, our sample consists of voluntarily admitted inpatients (those with sufficient insight into their symptoms and willingness to conceptualize their problems as signs of a mental disorder) and is only partially representative of the general population of people with MDD.

The third limitation is that the study was conducted in a single center. Although this center is the largest in Moscow specializing in free care for patients with non-psychotic mental disorders, we cannot exclude the possibility that data from other centers may be different.

The fourth limitation is the exclusion of MDD patients with psychotic symptoms. However, the variables associated with suicidality in these patients may differ substantially from those important in patients without psychotic features and may include several disease-specific factors, such as hallucinations and delusions. In this respect, the variables associated with suicidality in our study cannot be directly extrapolated to MDD patients with psychotic symptoms.

## Conclusion

The prevalence of suicidality among Russian inpatients with MDD during the pandemic is lower than that found in many pre-pandemic studies. Predictors of serious suicide risk in Russian patients with MDD during the pandemic are younger age, greater severity of state anxiety and depression, and NSSI. There was no association of suicidality in MDD with SARS-CoV2 infection, type of medical care for COVID-19 and any specific acute phase symptom.

## Data availability statement

The datasets presented in this article are not readily available due to the local legal restriction in regard to the psychiatric help utilization. Reasonable requests for access to the datasets should be directed to MZ, mzinchuk@mail.ru.

## Ethics statement

The study involved human subjects and its protocol was approved by the local research ethics committee of the Moscow Research and Clinical Center of Neuropsychiatry (protocol number 46). All study procedures were conducted in accordance with local legislation and institutional requirements. Written informed consent to participate in this study was obtained from all participants prior to study procedures.

## Author contributions

MZ: Conceptualization, Methodology, Project administration, Writing – original draft. GK: Data curation, Writing – original draft. MB: Writing – original draft. YB: Investigation, Writing – original draft. ES: Investigation, Writing – original draft. SP: Investigation, Writing – original draft. NV: Investigation, Writing – original draft. MT: Investigation, Resources, Writing – original draft. AY: Data curation, Formal Analysis, Writing – original draft. AG: Supervision, Writing – review & editing.
